# Noncausal effects between tea intake and migraine risk: a Mendelian randomization study

**DOI:** 10.1038/s41598-023-40171-z

**Published:** 2023-08-09

**Authors:** Chen Jin, Sijing Tu, Siyi Sun, Zhongyi Zhang, Xiaohe Wang

**Affiliations:** School of Public Health, Hangzhou Normal University, Hangzhou, 311121 China

**Keywords:** Health care, Risk factors

## Abstract

Observational studies have yielded conflicting results regarding the relationship between tea intake and migraine risk. Residual confounders and potential reverse causality are unavoidable in traditional observational studies. To provide evidence for establishing viable disease screening and prevention strategies, a Mendelian randomization study (MR) was conducted to determine the causal inference between tea intake and migraine. We obtained 28 single-nucleotide polymorphisms (SNPs) for any migraine (AM), 25 SNPs for migraine with aura (MA), and 27 SNPs for migraine without aura (MO) associated with tea intake derived from a large genome-wide association study (GWAS) of the UK Biobank (UKBB) (containing 447,485 samples). The largest migraine GWAS performed by the International Headache Genetics Consortium (IHGC), including 29,209 cases and 172,931 controls, provided data on migraines and their subtypes (MA and MO). We used the method of inverse variance weighting (IVW) with fixed effects as the first-string MR selection. Sensitivity analysis and MR-pleiotropy residual sum and outlier (MR-PRESSO) method further assessed the robustness of the findings. Based on the conclusion of IVW in the fixed effects model, we found that tea intake had no causal relationship with AM risk (odds ratio (OR), 0.94; 95% confidence interval (CI), 0.70–1.25; P = 0.65), MA risk (OR, 0.93; 95% CI, 0.51–1.72; P = 0.83), or MO risk (OR, 0.90; 95% CI, 0.52–1.54; P = 0.69). Sensitivity analyses and MR-PRESSO showed no directional pleiotropy or heterogeneity. Our two-sample MR investigation found no causality between tea intake and migraine risk in European populations, implying that attempts to change tea drinking habits may not lead to a reduced risk of migraine.

## Introduction

Migraine, a very disturbing headache, affects more than one billion individuals worldwide severely impacting the quality of life and hence remains a major global public health challenge^1,2^ and the leading cause of disability in several countries^3^. Therefore, determining influencing factors is key to migraine prevention and treatment.

Tea is one of the most common beverages in life and contains several biologically active ingredients, such as caffeine, polyphenols, tannins, vitamins, and saponins^4,5^. Caffeine from tea intake has been previously suggested to cause migraine^6–8^, and tea intake is a risk factor for syncope in migraineurs^9^. However, evidence also suggests that caffeine has a good analgesic effect to reduce pain when migraines occur^10^. The inconsistent and paradoxical relationship between tea intake and migraine in traditional epidemiological studies suggests that the causal relationship may be influenced by confounders and reverse causation. Therefore, whether tea intake induces migraine or migraineurs can reduce their pain by drinking tea remains nebulous; hence, clarifying the causality between these two factors may help to better facilitate the development of migraine prevention strategies. However, a better method is warranted to assess causality.

Mendelian randomization (MR) is an epidemiological method used to assess causality in exposure-outcome associations^11^ using genetic variants as instrumental variables (IVs) to measure exposures and prevent potential biases or reverse causality unavoidable in traditional observational studies. In the absence of conditions for randomized clinical trials (RCTs), the MR design provides new avenues for exploring causality since genetic variants are randomly assigned during meiosis, a process that simulates RCT. In this study, we used genetic variants linked to tea intake as IVs to investigate the causality of tea intake on migraine (AM), migraine with aura (MA), and migraine without aura (MO).

## Methods

### Study design

**Figure 1:**
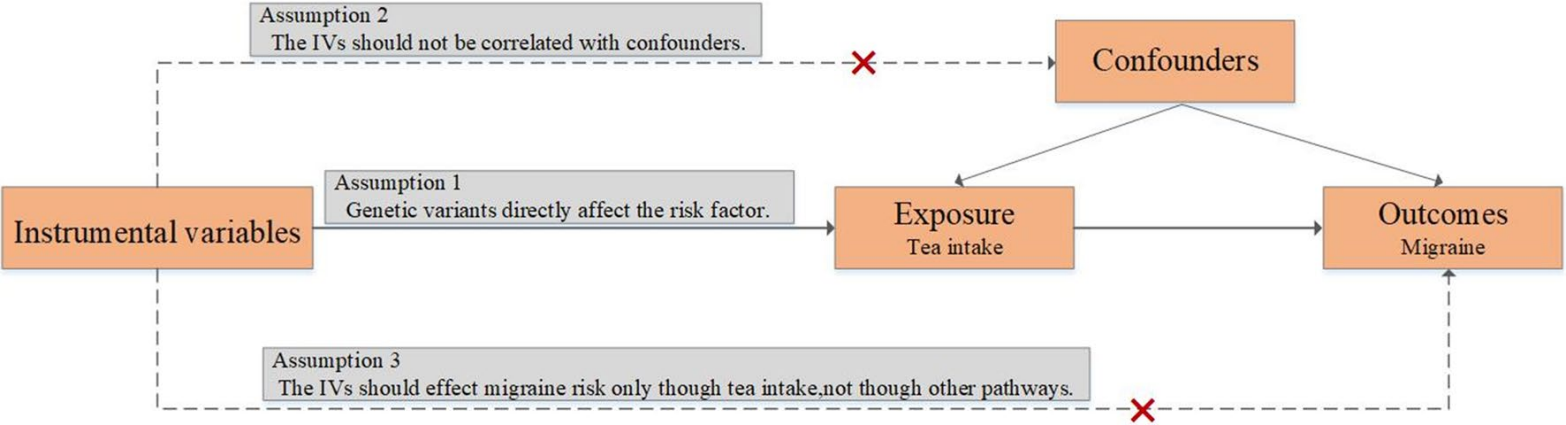
Schematic diagram showing the assumptions of Mendelian randomization analysis.

We employed a two-sample MR design to investigate the causal inference between tea intake and the risk of migraine development. Figure 1 illustrates the framework schematic of the MR concept connotation. The MR design is in line with the following three assumptions: (1) IVs (SNPs) are powerfully connected (P < 5 × 10^−8^) with tea intake; (2) IVs are not associated with underlying confounders; (3) IVs impact the outcome (migraines) only through exposure^12^. Our study protocol was in line with the STROBE-MR Statement, a guideline for reporting MR assay (Table S1)^13^. All methods were performed in accordance with the relevant guidelines and regulations.

### Data sources

MR analysis was performed using published summary-level data (Table 1). Specific ethical approval and informed consent were not required.

**Table 1:** Detailed information of data used for analyses.

Phenotype	Data source	Ethnicity	Sample size	Cases	Use in this MR study
Tea intake	IEU ID:ukb-b-6066	European	447,485	/	Exposure
Any migraine	IHGC	European	202,140	29,209	Outcome
Migraine with Aura			151,215	6332	
Migraine without Aura			147,970	8348	

IHGC, the International Headache Genetics Consortium.

We used heritable IVs of tea intake from the UK Biobank (UKBB), which included 447,485 samples (Phenotype Code:1488_raw; genome-wide association study (GWAS) ID ukb-b-6066). Specifically, the UKBB is a cohort study that involved participants from the UK aged 40–69 years^14^. Data on tea drinking habits were acquired via the questionnaire entry: “How many cups of tea do you drink each day? (Include black and green tea).” The reported tea intake ranged from 0 to 99 cups/day (mean and standard deviation, 3.51 ± 2.85 cups/day). Detailed information can be found on the IEU website (https://gwas.mrcieu.ac.uk/datasets/ukb-b-6066/). The GWAS meta-analysis comprised 22 studies with a total of 29,209 cases and 172,931 control subjects of European descent, conducted by the International Headache Genetics Consortium (IHGC), and provided the GWAS summary statistics on migraine used in this study^15^. The previously described diagnostic criteria of the International Headache Society clarified the definition of migraine^16^. Migraine was divided into the subtypes MA (144,883 controls and 6332 cases) and MO (139,622 controls and 8,348 cases) (Table 1).

### Selection of IVs

IVs were sorted using the following steps during our two-sample MR study: First, we obtained a total of 41 SNPs prominently associated with tea intake and served as IVs (P < 5 × 10^−8^). We then removed SNPs with horizontal polymorphic effects to satisfy the second key assumption by searching an online tool (the PhenoScanner database). Eight SNPs associated with potential confounders in coffee consumption cups per day or body mass index (P < 5 × 10^–8^) were removed (Table S2)^17^. We excluded no SNPs by linkage disequilibrium (r^2^ < 0.001; region size, 10,000 kb) and performed MR Steiger filtering tests to remove SNPs that suggested reverse causality to meet the third key assumption, excluding the restriction assumption (Table S3)^18^. F-statistics were calculated to determine the strength of genetic variation and SNPs with F-statistics > 10 were selected, indicating sufficient strength^19^. All candidate SNPs were found in the outcome datasets; hence, no proxy SNPs were required; 28 SNPs for AM (25 SNPs for MA and 27 SNPs for MO) were associated with tea intake as IVs (Table S4).

### Statistical analysis

We used several MR approaches to estimate each instrumental SNP in migraines, namely, the inverse variance weighted (IVW), weighted median, and MR-Egger, after harmonizing the SNPs across the GWASs of exposures and outcomes. The IVW method was adopted for the primary analysis to derive the final effect estimates^20^. The heterogeneity, pleiotropy, and leave-one-out sensitivity tests were combined into an MR sensitivity analysis. Horizontal pleiotropy indicates that the MR-Egger results provide a more reliable causal prediction^21^.

The MR pleiotropy residual sum and outlier (MR-PRESSO) method was used to detect horizontal pleiotropic outliers containing three elements^22^. The MR-PRESSO global test was used to detect horizontal pleiotropy. The MR-PRESSO outlier test identifies outliers (P < 0.05) and estimates the corrected results. The MR-PRESSO distortion test was used to determine significant differences before and after removing outlier correction. Statistical power was determined using the web software (https://shiny.cnsgenomics.com/mRnd/)^23^. Statistical analyses were performed using the packages of two-sample MR (version 0.5.6) and MR-PRESSO (version 1.0) in the R program (version 4.2.2).

### Ethics statement

The studies involving human participants were reviewed and approved by Local Ethics Committees of the UK Biobank project, the International Headache Genetics Consortium (IHGC). No additional ethical approval are required. The present MR analyses were approved by IHGC Publication Committee.

## Results

Table S3 shows the results of MR Steiger filtering tests. The IVs were estimated to explain 2.55% of the observed variance in tea intake; the F-statistics of all selected SNPs were much greater than the threshold of 10, which indicates that the tools used in our study have good strength (Table S4). We found no overlap in samples between the data sources of tea intake and migraine (Table 1).

**Figure 2:**
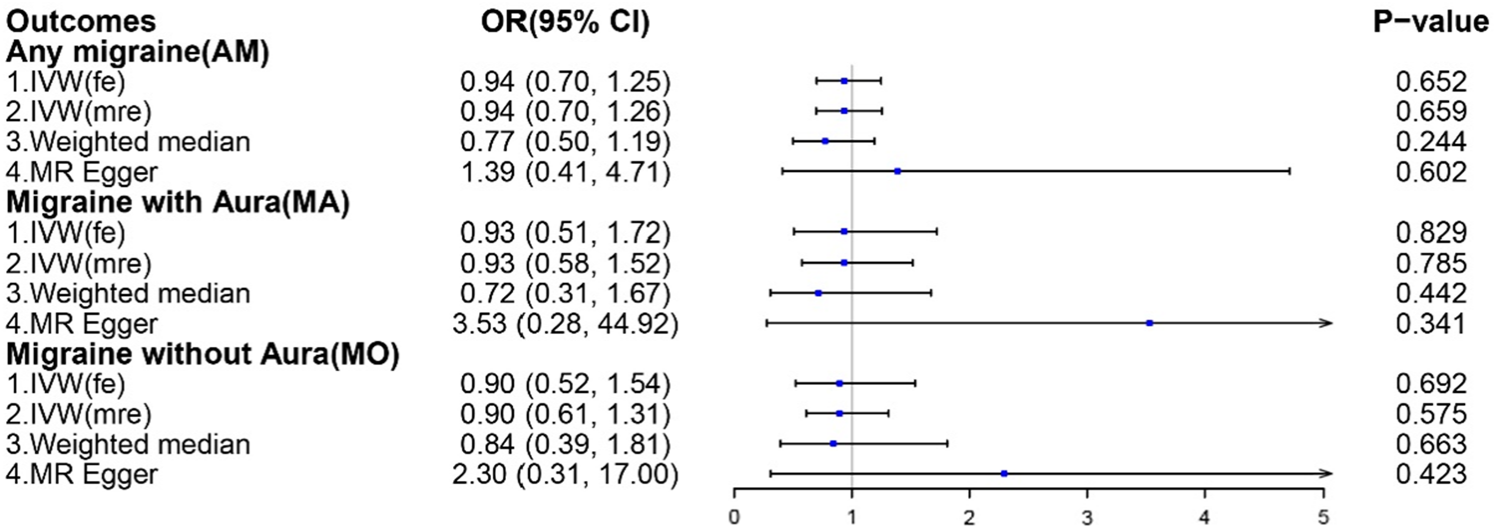
MR estimates of the associations between tea intake and migraines. IVW (fe), fixed-effects inverse-variance weighted; IVW (mre), multiplicative random-effects inverse-variance weighted; MR-Egger, Mendelian randomization-Egger.

**Figure 3:**
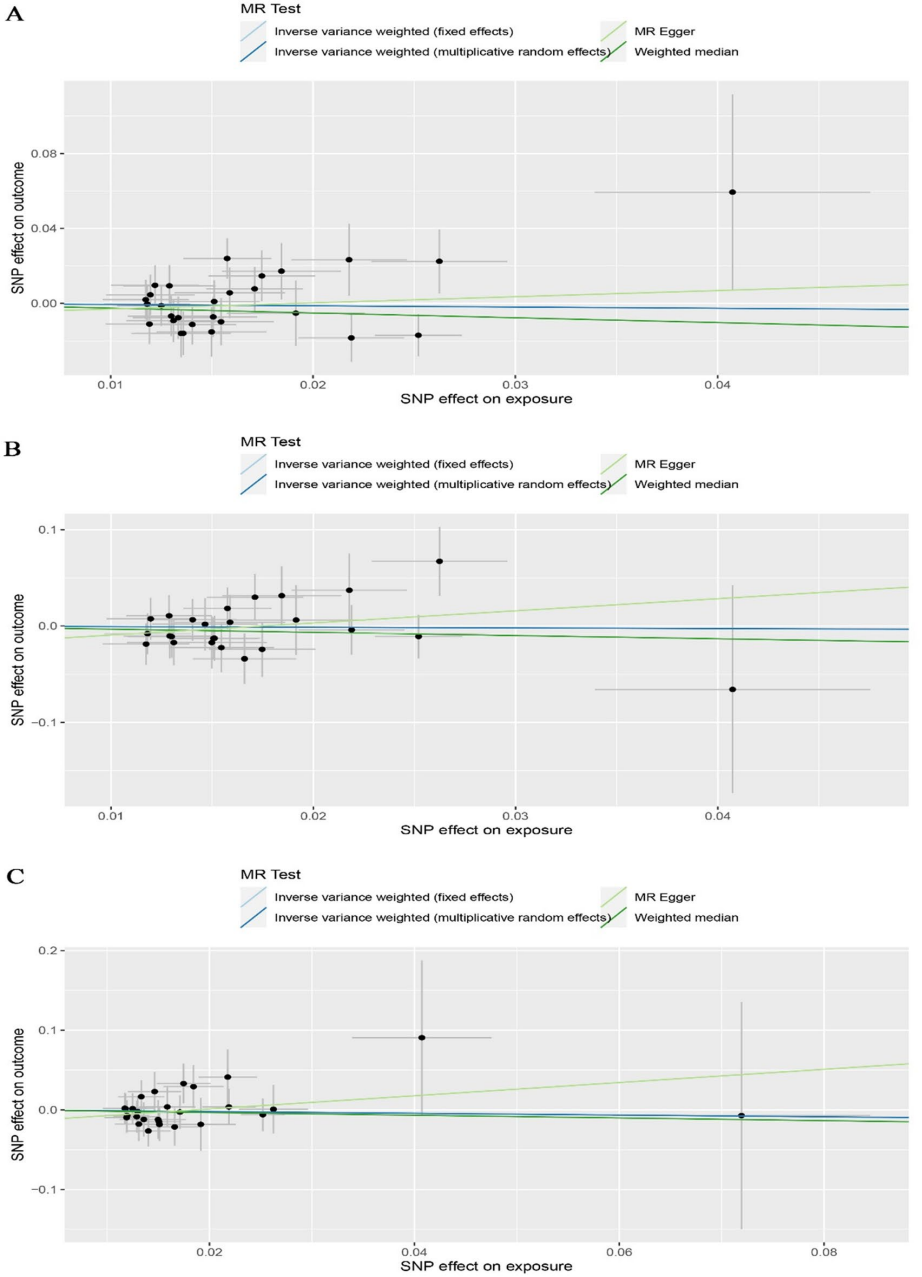
Scatter plot of SNPs related to tea intake and the migraine risk. (**A**) Scatter plot of tea intake-AM risk MR. (**B**) Scatter plot of tea intake-MA risk MR. (**C**) Scatter plot of tea intake-MO risk MR. AM, any migraines; MA, migraines with aura; MO, migraines without aura; SNP, single-nucleotide polymorphism.

The main analytical method, IVW, showed no significant causal effects between tea intake and AM, MA, or MO risk. The odds ratio (OR) of tea intake and migraines were as follows: AM OR, 0.94 (95% confidence interval (CI), 0.70–1.25); MA OR, 0.93 (95% CI, 0.51–1.72); MO OR, 0.90 (95% CI, 0.52–1.54) (Fig. 2). Despite the MR-Egger regression presenting a wider CI, the results of the weighted median and MR-Egger regressions were consistent. The scatter plots of the association estimates between tea intake and AM, MA, and MO are shown in Fig. 3, while the forest plots of single SNPs affecting the risk of AM and its subtypes are shown in Fig. S1.

The MR-Egger intercept analysis and MR-PRESSO global test showed no horizontal pleiotropy between tea intake and AM subtype (Table S5). The funnel plots (Fig. S2) and the results of Cochran Q-derived p, I^2^ demonstrated by the IVW analyses (Table S5), further showed no heterogeneity. The leave-one-out plots for the MR analyses of tea intake for AM,MA, and MO indicated that the noncausal relationship was not altered by any single SNP (Fig. S3).

## Discussion

In this study, we found consistent evidence of the noncausal association between tea intake and migraine. To the best of our knowledge, this is the first study that has implemented an MR design to explore the causal relationship between tea consumption and migraine risk.

Previous studies have been based on clinical observations, without establishing absolute causality connections. A previous randomized case–control study reported a positive association between tea intake and migraine (P < 0.0001)^24^, while a large cross-sectional study has showed that high tea intake was associated with an increased prevalence of headaches (OR, 1.1; 95% CI, 1.01–1.2; P = 0.02)^25^. Furthermore, tea intake is associated with an increased risk of syncope in migraine patients (OR, 1.84; 95% CI, 1.22–2.79; P = 0.004)^9^. In contrast, a double-blind, randomized control trial reported that a fixed combination of caffeine was more effective for the treatment of headaches^26^.

Our results on tea intake, do not indicate a cause-and-effect relationship with migraine, possibly due to the following reasons: First, the false causal link between tea intake and migraine may stem from the fact that people distract themselves from migraine attacks by craving food. Second, a significant increase in tea intake among migraineurs may be attributed to the belief that caffeine is an analgesic adjuvant that relieves headaches, hence the erroneous conclusions in traditional studies. Positive results in observational studies may be attributed to residual confounders or reverse causality.

The major merit of our study was that the MR design reduces residual confounders and other biases, thereby strengthening the inference of causality and allowing for robust estimates of causality between tea intake and migraine outcomes. The two-sample MR study provided a large sample size, overcoming the difficulties and biases of cohort studies.

This study has some limitations. First, our research was restricted to participants of European descent; therefore, further investigation are warranted to confirm our findings in other ethnicities. Second, although we used methods such as sensitivity analysis to detect the level of pleiotropy and heterogeneity, we could not determine the likelihood of the selected tea intake-associated SNPs breaching the MR assumption. Third, 2.55% of the observed variance in tea intake was accounted for SNPs, possibly leading to insufficient statistical power. Finally, since the data on tea intake were self-reported, there may be some measurement bias in this study. In addition, genetic instruments are selected using statistical, rather than biological methods, and therefore may not be specific for tea drinking, which may result in the heritability of tea drinking not being very high, ultimately reducing the clinical significance of our MR analysis.

## Conclusion

The present MR analysis provided genetic evidence, stronger than that of previous observational studies, of no causality between tea intake and migraine risk in individuals of European ancestry. Our findings suggest that changing tea drinking habits might not be an effective strategy for reducing migraine risk.

### Supplementary Information

Supplementary Tables.

Supplementary Information 2.

## Data Availability

The original contributions presented in the study are included in the article/Supplementary Material, further inquiries can be directed to the corresponding author.

## Abbreviations

SNPs: Single-nucleotide polymorphisms
AM: Any migraine
MA: Migraine with aura
MO: Migraine without aura
GWAS: Genome-wide association study
UKBB: UK Biobank
IHGC: International Headache Genetics Consortium
IVW: Inverse variance weighting
MR-PRESSO: MR-pleiotropy residual sum and outlier method
OR: Odds ratio
CI: Confidence interval
IVs: Instrumental variables
RCTs: Randomized clinical trials
