# Supplementary material for: Noncausal effects between tea intake and migraine risk: a Mendelian randomization study
Source: Sci Rep. 2023 Aug 9;13:12898. doi: 10.1038/s41598-023-40171-z (PMC10412590; doi:10.1038/s41598-023-40171-z)
Supplement: Supplementary file 2 — Supplementary Information 2. [file 41598_2023_40171_MOESM2_ESM.docx]

**Figure S1.** Forest plots of a single SNP effect on the risk of any migraine and its subtypes.


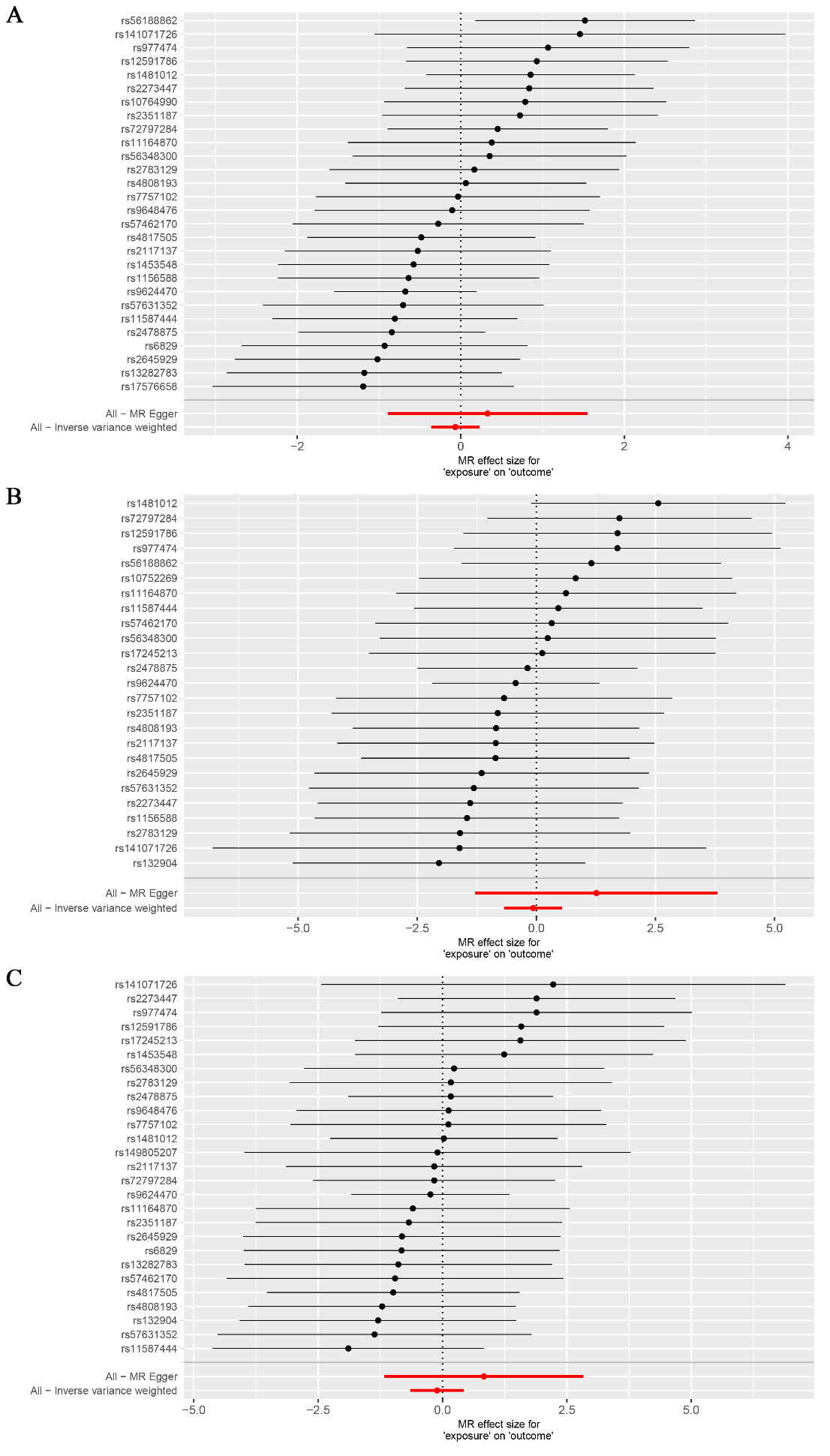


**Figure S2.** Funnel plots of genetic associations between tea intake and causal estimates of any migraine (A), migraines with aura (B), and migraines without aura (C).


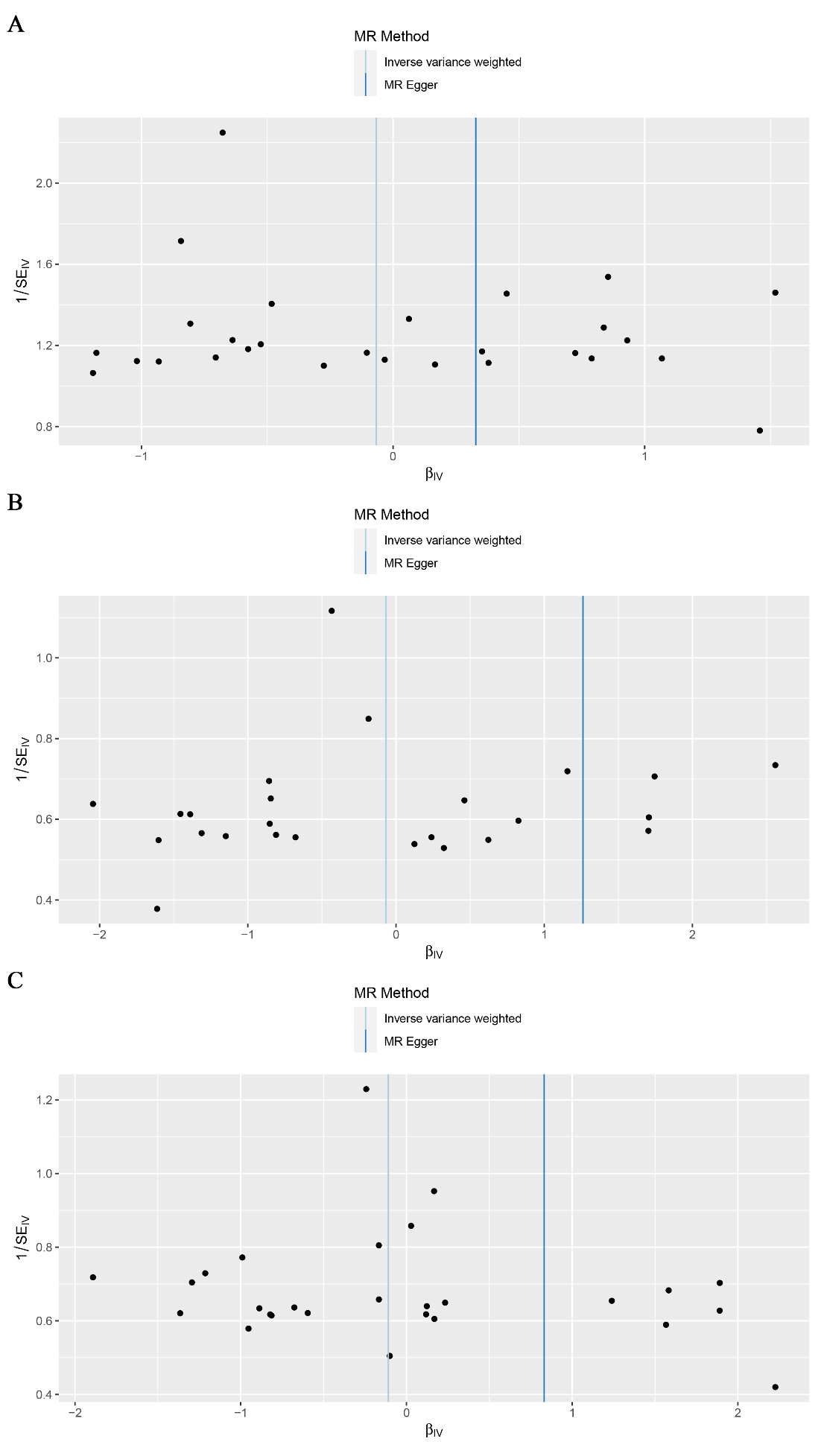


**Figure S3.** Leave-one-out plots for the MR analyses of tea intake on any migraine and its subtypes


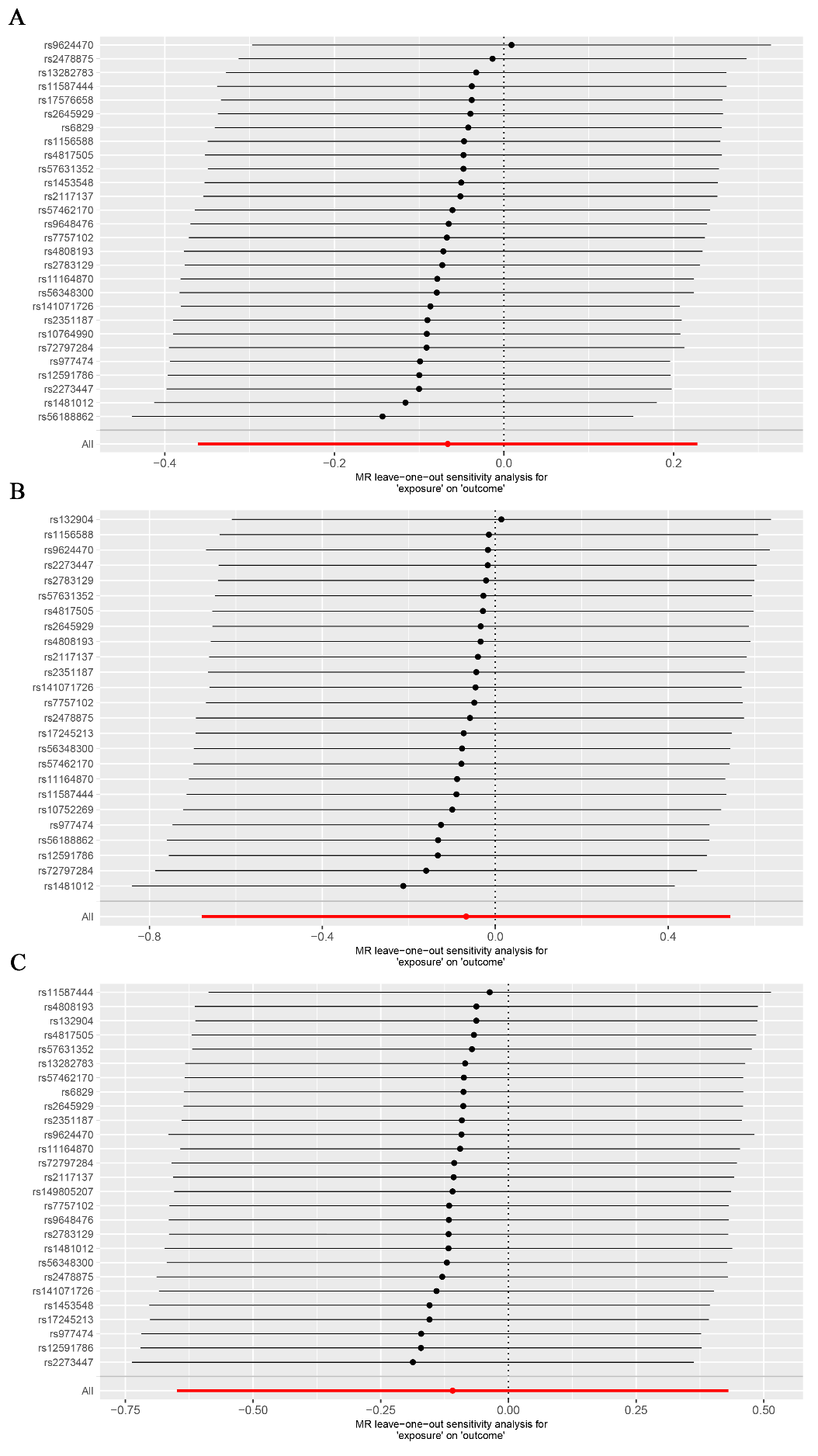


**International Headache Genetics Consortium Members**

Padhraig Gormley^31 34^, Verneri Anttila^32,33,35^, Bendik S. Winsvold^36 38^, Priit Palta^39^, Tonu Esko^32,40,41^, Tune H. Pers^32,41 43^, Kai-How Farh^32,35,44^, Ester Cuenca- Leon^31 33,45^, Mikko Muona^39,46 48^, Nicholas A. Fur- lotte^30^, Tobias Kurth^49,9^, Andres Ingason^10^, George McMahon^50^, Lannie Ligthart^51^, Gisela M. Terwindt^52^, Mikko Kallela^53^, Tobias M. Freilinger^54,55^, Caroline Ran^56^, Scott G. Gordon^22^, Anine H. Stam^52^, Stacy Steinberg^10^, Guntram Borck^57^, Markku Koiranen^58^, Lydia Quaye^59^, Hieab H. H. Adams^6,61^, Terho Lehtim€aki^62^, Antti-Pekka Sarin^39^, Juho Wedenoja^63^, David A. Hinds^30^, Julie E. Bur- ing^9,64^, Markus Schu€rks^65^, Paul M. Ridker^9,64^, Maria Gud- laug Hrafnsdottir^66^, Hreinn Stefansson^10^, Susan M. Ring^50^, Jouke-Jan Hottenga^51^, Brenda W. J. H. Penninx^67^, Markus F€arkkil€a^53^, Ville Artto^53^, Mari Kaunisto^39^, Salli Veps€al€ainen^53^, Rainer Malik^55^, Andrew C. Heath^68^, Pamela A. F. Madden^68^, Nicholas G. Martin^22^, Grant W. Montgomery^8^, Mitja I. Kurki^31 33,39,69^, Mart Kals^40^, Reedik M€agi^40^, Kalle P€arn^40^, Eija H€am€al€ainen^39^, Hailiang Huang^32,33,35^, Andrea E. Byrnes^32,33,35^, Lude Franke^70^, Jie Huang^34^, Evie Stergiakouli^50^, Phil H. Lee^31 33^, Cynthia Sandor^71^, Caleb Webber^71^, Zameel Cader^72,73^, Bertram Muller-Myhsok^74,75^, Stefan Schreiber^76^, Thomas Meitinger^77,78^, Johan G. Eriksson^79,8^, Veikko Salomaa^80^, Kauko Heikkil€a^81^, Elizabeth Loehrer^60,82^, Andre G. Uitter- linden^83^, Albert Hofman^60^, Cornelia M. van Duijn^60^, Lynn Cherkas^59^, Linda M. Pedersen^36^, Audun Stubhaug^84,85^, Christopher S. Nielsen^84,86^, Minna M€annikko€^58^, Evelin Mihailov^40^, Lili Milani^40^, Hartmut Go€bel^87^, Ann-Louise Esserlind^88^, Anne Francke Christensen^88^, Thomas Folk- mann Hansen^89^, Thomas Werge^90,91,7^, Jaakko Kaprio^39,63,92^, Arpo J. Aromaa^80^, Olli Raitakari^93,94^, M. Arfan Ikram^60,61,95^, Tim Spector^59^, Marjo-Riitta J€arvelin^58,96 98^, Andres Metspalu^40^, Christian Kubisch^99^, David P. Strachan^100^, Michel D. Ferrari^52^, Andrea C. Belin^56^, Martin Dichgans^55,75^, Maija Wessman^39,46^, Arn M. J. M. van den Maagdenberg^52,101^, John-Anker Zwart^36^ ^38^, Dorret I. Boomsma^51^, George Davey Smith^50^, Kari Stefansson^10,102^, Nicholas Eriksson^30^, Mark J. Daly^32,33,35^, Benjamin M. Neale^32,33,35^, Jes Olesen^88^, Daniel I. Chasman^9^, Dale R. Nyholt^1^, Aarno Palotie^31,35,103^.

**International Headache Genetics Consortium Affiliations**

^1^School of Biomedical Sciences, Faculty of Health, and Institute of Health and Biomedical Innovation, Queens- land University of Technology, Brisbane, Queensland, Australia. ^2^Department of Epidemiology and Cancer Con- trol, St. Jude Children’s Research Hospital, Memphis, Tennessee 38105, USA. ^3^23andMe, Inc., 899 W. Evelyn Avenue, Mountain View, California 94041, USA. ^4^School of Pharmacy and Biomedical Sciences, University of Cen- tral Lancashire, Preston PR1 2HE, United Kingdom. ^5^Department of Obstetrics and Gynecology, Niigata University Graduate School of Medical and Dental Sciences, Niigata 950-2181, Japan. ^6^Department of Biome- dicine - Human Genetics, Aarhus University, DK-8000 Aarhus, Denmark. ^7^iPSYCH, The Lundbeck Foundation Initiative for Integrative Psychiatric Research, DK-2100 Copenhagen, Denmark. ^8^Institute for Molecular Bio- science, The University of Queensland, Brisbane, Queens- land 4072, Australia. ^9^Divisions of Preventive Medicine, Department of Medicine, Brigham and Women’s Hospi- tal, Harvard Medical School, Boston, MA, USA. ^10^deCODE Genetics/Amgen, 101 Reykjavik, Iceland. ^11^Department of Biostatistics, University of Liverpool, Liverpool L69 3GL, UK. ^12^Wellcome Trust Centre for Human Genetics, University of Oxford, Oxford OX3 7BN, UK.^13^KULeuven, Department of Development and Regeneration, Organ systems, 3000 Leuven, Belgium. ^14^Department of Obstetrics and Gynaecology, Leuven University Fertility Centre, University Hospital Leuven, 3000 Leuven, Belgium. ^15^Harvard T.H. Chan School of Public Health, Boston, Massachusetts 02115, USA. ^16^Channing Division of Network Medicine, Department of Medicine, Brigham and Women’s Hospital and Har- vard Medical School, Boston, Massachusetts 02115, USA. ^17^Division of Preventive Medicine, Brigham and Women’s Hospital, Boston, Massachusetts 02215, USA. ^18^Institute of Medicine and Public Health, Vanderbilt University Medical Center, Nashville, Tennessee 37203, USA. ^19^Van- derbilt Genetics Institute, Division of Epidemiology, Insti- tute of Medicine and Public Health, Department of Medicine, Vanderbilt University Medical Center, Nash- ville, Tennessee 37203, USA. ^20^Cognitive Science Depart- ment, University of California, San Diego, La Jolla, California 92093, USA. ^21^Institute of Biological Psychiatry, Mental Health Centre Sct. Hans, Copenhagen University Hospital, DK-2100 Copenhagen, Denmark. ^22^Department of Genetics and Computational Biology, QIMR Berghofer Medical Research Institute, Brisbane, Queensland 4006, Australia. ^23^Endometriosis CaRe Centre, Nuffield Dept of Obstetrics & Gynaecology, University of Oxford, John Radcliffe Hospital, Oxford OX3 9DU, UK. ^24^Center for Integrative Medical Sciences, RIKEN, Yokohama 230- 0045, Japan. ^25^Institute of Medical Sciences, The Univer- sity of Tokyo, Tokyo 108-8639, Japan. ^26^Department of Obstetrics and Gynecology, Landspitali University Hospi- tal, 101 Reykjavik, Iceland. ^27^Faculty of Medicine, School of Health Sciences, University of Iceland, 101 Reykjavik, Iceland. ^28^Vanderbilt Genetics Institute, Vanderbilt Epi- demiology Center, Institute of Medicine and Public Health, Department of Obstetrics and Gynecology, Van- derbilt University Medical Center, Nashville, Tennessee 37203, USA. ^29^Global Medical Affairs Fertility, Research and Development, Merck KGaA, Darmstadt, Germany. ^30^23andMe, Inc., 899 W. Evelyn Avenue, Mountain View, California 94041, USA. ^31^Psychiatric and Neurodevelop- mental Genetics Unit, Massachusetts General Hospital and Harvard Medical School, Boston, Massachusetts, USA. ^32^Medical and Population Genetics Program, Broad Institute of MIT and Harvard, Cambridge, Massachusetts, USA. ^33^Stanley Center for Psychiatric Research, Broad Institute of MIT and Harvard, Cambridge, Massachusetts, USA. ^34^Wellcome Trust Sanger Institute, Wellcome Trust Genome Campus, Hinxton, UK. ^35^Analytic and Transla- tional Genetics Unit, Massachusetts General Hospital and Harvard Medical School, Boston, Massachusetts, USA. ^36^FORMI, Oslo University Hospital, Oslo, Norway. ^37^Department of Neurology, Oslo University Hospital, Oslo, Norway. ^38^Institute of Clinical Medicine, University of Oslo, Oslo, Norway. ^39^Institute for Molecular Medicine Finland (FIMM), University of Helsinki, Helsinki, Fin- land. ^40^Estonian Genome Center, University of Tartu, Tartu, Estonia. ^41^Division of Endocrinology, Boston Chil- dren’s Hospital, Boston, Massachusetts, USA. ^42^Depart- ment of Epidemiology Research, Statens Serum Institut, Copenhagen, Denmark. ^43^Novo Nordisk Foundation Center for Basic Metabolic Research, University of Copenhagen, Copenhagen, Denmark. ^44^Illumina, San Diego, California, USA. ^45^Pediatric Neurology, Vall d’Hebron Research Institute, Barcelona, Spain. ^46^Folkh€al- san Institute of Genetics, Helsinki, Finland. ^47^Neuro- science Center, University of Helsinki, Helsinki, Finland. ^48^Molecular Neurology Research Program, Research Pro- grams Unit, University of Helsinki, Helsinki, Finland. ^49^Institute of Public Health, Charit'e–Universit€atsmedizin Berlin, Berlin, Germany. ^50^Medical Research Council (MRC) Integrative Epidemiology Unit, University of Bris- tol, Bristol, UK. ^51^Department of Biological Psychology, Vrije Universiteit, Amsterdam, the Netherlands. ^52^Depart- ment of Neurology, Leiden University Medical Centre, Leiden, the Netherlands. ^53^Department of Neurology, Hel- sinki University Central Hospital, Helsinki, Finland. ^54^Department of Neurology and Epileptology, Hertie- Institute for Clinical Brain Research, University of Tue- bingen, Tuebingen, Germany. ^55^Institute for Stroke and Dementia Research, Klinikum der Universit€at Mu€nchen, Ludwig-Maximilians-Universit€at Mu€nchen, Munich, Ger- many. ^56^Department of Neuroscience, Karolinska Insti- tutet, Stockholm, Sweden. ^57^Institute of Human Genetics, Ulm University, Ulm, Germany. ^58^Center for Life Course Epidemiology and Systems Medicine, University of Oulu, Oulu, Finland. ^59^Department of Twin Research and Genetic Epidemiology, King’s College London, London, UK. ^60^Department of Epidemiology, Erasmus University Medical Center, Rotterdam, the Netherlands. ^61^Depart- ment of Radiology, Erasmus University Medical Center, Rotterdam, the Netherlands. ^62^Department of Clinical Chemistry, Fimlab Laboratories, School of Medicine, University of Tampere, Tampere, Finland. ^63^Department of Public Health, University of Helsinki, Helsinki, Fin- land. ^64^Harvard Medical School, Boston, Massachusetts, USA. ^65^Department of Neurology, University Duisburg– Essen, Essen, Germany. ^66^Landspitali University Hospital, Reykjavik, Iceland. ^67^Department of Psychiatry, VU University Medical Centre, Amsterdam, the Netherlands. ^68^Department of Psychiatry, Washington University School of Medicine, St. Louis, Missouri, USA. ^69^Depart- ment of Neurosurgery, NeuroCenter, Kuopio University Hospital, Kuopio, Finland. ^70^Department of Genetics, University Medical Center Groningen, University of Groningen, Groningen, the Netherlands. ^71^MRC Func- tional Genomics Unit, Department of Physiology, Anat- omy & Genetics, Oxford University, Oxford, UK. ^72^Nuffield Department of Clinical Neuroscience, Univer- sity of Oxford, Oxford, UK. ^73^Oxford Headache Centre, John Radcliffe Hospital, Oxford, UK. ^74^Max Planck Insti- tute of Psychiatry, Munich, Germany. ^75^Munich Cluster for Systems Neurology (SyNergy), Munich, Germany. ^76^Institute of Clinical Molecular Biology, Christian Albrechts University, Kiel, Germany. ^77^Institute of Human Genetics, Helmholtz Zentrum Mu€nchen, Neuherberg, Germany. ^78^Institute of Human Genetics, Technische Universit€at Mu€nchen, Munich, Germany. ^79^Department of General Practice and Primary Health Care, University of Helsinki and Helsinki University Hospital, Helsinki, Finland. ^80^National Institute for Health and Welfare, Hel- sinki, Finland. ^81^Institute of Clinical Medicine, University of Helsinki, Helsinki, Finland. ^82^Department of Environ- mental Health, Harvard T.H. Chan School of Public Health, Boston, Massachusetts, USA. ^83^Department of Internal Medicine, Erasmus University Medical Center, Rotterdam, the Netherlands. ^84^Department of Pain Man- agement and Research, Oslo University Hospital, Oslo, Norway. ^85^Medical Faculty, University of Oslo, Oslo, Nor- way. ^86^Department of Ageing and Health, Norwegian Institute of Public Health, Oslo, Norway. ^87^Kiel Pain and Headache Center, Kiel, Germany. ^88^Danish Headache Center, Department of Neurology, Rigshospitalet, Glostrup Hospital, University of Copenhagen, Copen- hagen, Denmark. ^89^Institute of Biological Psychiatry, Mental Health Center Sct. Hans, University of Copen- hagen, Roskilde, Denmark. ^90^Institute of Biological Psy- chiatry, MHC Sct. Hans, Mental Health Services Copenhagen, Copenhagen, Denmark. ^91^Institute of Clini- cal Sciences, Faculty of Medicine and Health Sciences, University of Copenhagen, Copenhagen, Denmark. ^92^Department of Health, National Institute for Health and Welfare, Helsinki, Finland. ^93^Research Centre of Applied and Preventive Cardiovascular Medicine, Univer- sity of Turku, Turku, Finland. ^94^Department of Clinical Physiology and Nuclear Medicine, Turku University Hospital, Turku, Finland. ^95^Department of Neurology, Erasmus University Medical Center, Rotterdam, the Netherlands. ^96^Department of Epidemiology and Bio- statistics, MRC Health Protection Agency (HPE) Centre for Environment and Health, School of Public Health, Imperial College London, London, UK. ^97^Biocenter Oulu, University of Oulu, Oulu, Finland. ^98^Unit of Primary Care, Oulu University Hospital, Oulu, Finland. ^99^Institute of Human Genetics, University Medical Center Hamburg- Eppendorf, Hamburg, Germany. ^100^Population Health Research Institute, St George’s, University of London, London, UK. ^101^Department of Human Genetics, Leiden University Medical Centre, Leiden, the Net herlands. ^102^Faculty of Medicine, University of Iceland, Reykjavik, Iceland. ^103^Department of Neurology, Massachusetts Gen- eral Hospital, Boston, Massachusetts, USA.
